# A multicentre study on persistent atrial fibrillation ablation targeting rotational activity on top of pulmonary vein isolation: insights from an extensive mapping technique

**DOI:** 10.3389/fcvm.2026.1746001

**Published:** 2026-04-10

**Authors:** Marco Scaglione, Francesco Geuna, Enrico Guido Spinoni, Andrea Lamanna, Francesca Bulian, Francesco Solimene, Ricarda Marinigh, Vincenzo Schillaci, Alberto Battaglia, Alberto Arestia, Maurizio Del Greco, Marco Gagliardi, Massimiliano Maines, Giulio Zucchelli, Francesco Pentimalli, Natascia Cerrato, Giuseppe Stabile, Giuseppe Allocca, Domenico Caponi, Roberto Mantovan

**Affiliations:** 1 Division of Cardiology, Cardinal Massaia Hospital, Asti, Italy; 2 Johnson & Johnson Medical S.p.A., Pomezia, Italy; 3 Division of Cardiology, Conegliano Hospital Ulss2, Conegliano, Italy; 4 Division of Cardiology, Montevergine Clinic, Mercogliano, Italy; 5 Division of Cardiology, Santa Maria del Carmine, Rovereto, Italy; 6 Division of Cardiology, Azienda Ospedaliera Universitaria di Pisa, Pisa, Italy; 7 Division of Cardiology, San Paolo Hospital, Savona, Italy; 8 Division of Cardiology, Anthea Hospital, Bari, Italy; 9 Division of Cardiology, Mediterranea Cardiocentro, Napoli, Italy

**Keywords:** CARTO finder, persistent atrial fibrillation, pulmonary veins isolation, radiofrequency ablation, rotational activity, rotor

## Abstract

**Background-aims:**

Recently, the etiopathogenetic role of rotational activity (RotAct) in atrial fibrillation (AF) has been proposed. We designed a prospective multicenter study using CARTO Finder to evaluate the presence/distribution of RotActs in persistent AF (persAF), pulmonary vein isolation (PVI)influences on RotActs, and the impact of its elimination on top of PVI on procedural outcomes.

**Methods:**

For this study, 76 patients with pers AF ablation were enrolled. Procedural steps involved (1) using a CARTO-Finder map to look for RotActs (physician blinded); (2) PVI; (3) using a new map to look for residual/new RotActs; (4) ablation of RotActs, if present; (5) and finally using a new map to confirm RotAct elimination. Populations were divided based on the presence or absence of structural heart disease (Group I and II) and the presence or absence (R+ and R−) of RotActs before PVI. Presence, number, and distribution of RotAct at STEP 1, the impact of PVI on RotAct at STEP 3, and maintenance of sinus rhythm (SR) during follow-up were evaluated. 56 AF patients undergoing standard ablation protocol were included as a control group.

**Results:**

RotAct was identified in 29 (38%) patients at STEP1. RotAct did not differ between GI and GII patients. PVI significantly modified the number and localization of RotActs (*p* = 0.012). RotActs which were present at STEP 1 were different after PVI, with disappearance in 18 and new appearance in 5 patients. 71 patients completed a mean 13 ± 6 months follow-up and 91.5% were in stable SR. No difference in relapses was seen between R + and R- and GI and GII. RotAct ablation significantly reduced arrhythmia relapse during the follow-up compared to the control group (freedom from arrhythmia 91.5% vs. 78.6%, *p* = 0.025).

**Conclusion:**

RotAct was present in 38% in persAF patients. PVI influenced the number and distribution of RotActs. A tailored ablation strategy provided a high success rate (91.5%) at the follow-up.

## Introduction

Atrial fibrillation (AF) is the most common supraventricular arrhythmia, having an important clinical impact in terms of morbidity, mortality, and quality of life (1). Antiarrhythmic drugs (AADs) showed limited efficacy in the prevention of AF recurrence and, consequently, transcatheter ablation has been developed, demonstrating very good results. Pulmonary vein isolation (PVI) has become a standard procedure after the demonstration of PV focal activity as a trigger for AF (2). The success rate of PVI in reducing AF recurrence is higher in paroxysmal compared to persistent AF (persAF) patients (3–6). The reason for this behavior could be related to a greater substrate remodeling in the etiopathogenesis of the latter form (7–9). To improve the success rate in this latter group of patients, different ablation strategies have been proposed, such as CFAE ablation (10, 11), adjunctive linear lesions (left isthmus and left atrial roof line) (12, 13), and the creation of a posterior box (14, 15), showing conflicting results. The STAR AF II trial was designed to identify a better ablation strategy, demonstrating no statistically significant difference in the ablation outcomes comparing PVI only, PVI plus CFAE ablation, and/or linear lesions. All three groups demonstrated a similar success rate of about 60% (16).

Recently, pulsed field ablation (PFA) has been introduced as an effective and safe energy modality in AF ablation. However, while PFA has yielded optimal freedom from recurrence in paroxysmal AF, available data for persistent forms indicate a limitation in efficacy, comparable to that observed with radiofrequency and cryo-energy (17–19). These data underscore the importance of identifying the etiopathological drivers in patients with persAF to improve success rates in this patient population, particularly for those who do not respond to standard PVI ablation approach.

Narayan et al. postulated the involvement of rotational activity (RotAct) in the genesis and maintenance of persAF and proposed a new device to map the atrium to detect these activities (20). However, the promising results obtained by this author have not been reproduced by others (21). In addition, this technique required a dedicated system with a complex algorithm (22, 23).

Therefore, the importance of RotAct in the etiopathogenesis of AF and its role as a target in the ablation of persAF ablation is still uncertain.

The mapping software *CARTO Finder* embedded in the CARTO3 System (*Biosense Webster, Irvine, California, USA*) using a standard mapping catheter (*Pentaray* or *Octaray, Biosense Webster, Irvine, California, USA*) has been used to identify areas having rotational and focal activity during AF.

Based on the above-mentioned considerations, we decided to design a prospective multicenter study using the CARTO Finder software to (1) evaluate the presence and distribution of RotAct in persAF, (2) evaluate the influence of PVI on this activity, (3) assess the impact on the outcome of additional linear lesions aiming to eliminate RotAct on top of PVI in patients with persAF, and (4) compare with a persAF consecutive cohort undergoing “standard” ablation strategy.

## Methods

### Study population and data collection

This prospective multicenter study involves seven Italian electrophysiology laboratories with extensive experience in AF transcatheter ablation, coordinated by Cardinal Massaia Hospital in Asti, Italy.

76 consecutive patients with symptomatic pers AF were enrolled in the study from October 2020 to March 2022 and underwent transcatheter AF ablation using the CARTO3 mapping system. Pers AF was defined as an episode of AF lasting more than one week and less than one year (1).

A consecutive cohort of 56 patients with PersAF undergoing “standard” transcatheter ablation of AF using the CARTO3 mapping system were prospectively included in the analysis. The ablation procedure was performed from October 2020 to March 2022 and PVI plus adjunctive ablation lesions (posterior wall, roof, mitral isthmus, anterior wall) was applied to all patients according to the physician’s choice.

Inclusion criteria were

- Age older than 18 years old;
- First procedure of transcatheter ablation of Pers AF.

Exclusion criteria were

- Presence of SR at the time of the ablation procedure;
- Age below 18 years;
- Inability or unwilling to provide informed consent;
- Previous AF ablation procedure (no re-do procedures);
- Severe valvular defects and/or with programmed cardiac surgical intervention.

At the time of the patient admission, complete revision and collection of the clinical history was performed by the study investigators, including detailed information on symptoms, timing of AF onset, pharmacological therapy (if any), and transthoracic echocardiography on shared database. During the hospital stay, information on the ablation procedure was collected by the study investigators in each participating center.

After hospital discharge, follow-up was planned as follows:

Ambulatory evaluation including physical examination, ECG, and 24-hours ECG Holter monitoring were planned at 3-months after the ablation and then every 6 months or in case of symptoms. All these data were collected by study investigators in the shared database.

In case of relapse of AF during the first three months blanking period after ablation, electrical cardioversion was performed to restore sinus rhythm; this was not considered a failure of the procedure.

### Study endpoints

Primary endpoints of the present study were

- the evaluation of the presence and distribution of RotAct in patients with persAF;
- the influence of PVI on RotAct;
- sinus rhythm maintenance with or without AADs during the follow-up (at least 6 months) of a tailored ablation strategy including RotAct ablation on top of PVI;
- comparison of sinus rhythm maintenance between the study group (RotAct ablation) and the control group (“standard” ablation protocol) and the subgroups [Group (R+) vs. (R−) and vs. “standard” ablation protocol group] during the follow-up.

### Rotational activity definition

RotAct was defined by the CARTO Finder Algorithm as follows: a distance of less than 20 mm between the first spline receiving the electrical signal and the last one, a total temporal interval between the first signal identified and the last one covering at least the 50% of the identified cycle length (CL), maintained for two consecutive cycles on four of 5 splines. Every acquisition had to last at least 30 s, with TPI (Tissue Proximity Index) active (Supplementary Video 1).

### Ablation procedure

For the ablation procedure, a traditional fluoroscopic view and the navigation system CARTO3 EAM (Electro Anatomic Mapping) (Biosense Webster, Irvine, CA) was used. The procedure was performed via both right and left femoral veins.

A decapolar electrode catheter was positioned in the coronary sinus [Decanav (BiosenseWebster, Irvine, CA) via a transseptal puncture or a patent foramen ovale (PFO), if present. The left atrium (LA) was reached and the ablation catheter [Thermocool SF Smart-touch catheter (Biosense Webster Inc, CA) was inserted. EAM reconstruction was performed in every patient using the Pentaray mapping catheter.

**Figure 1:**
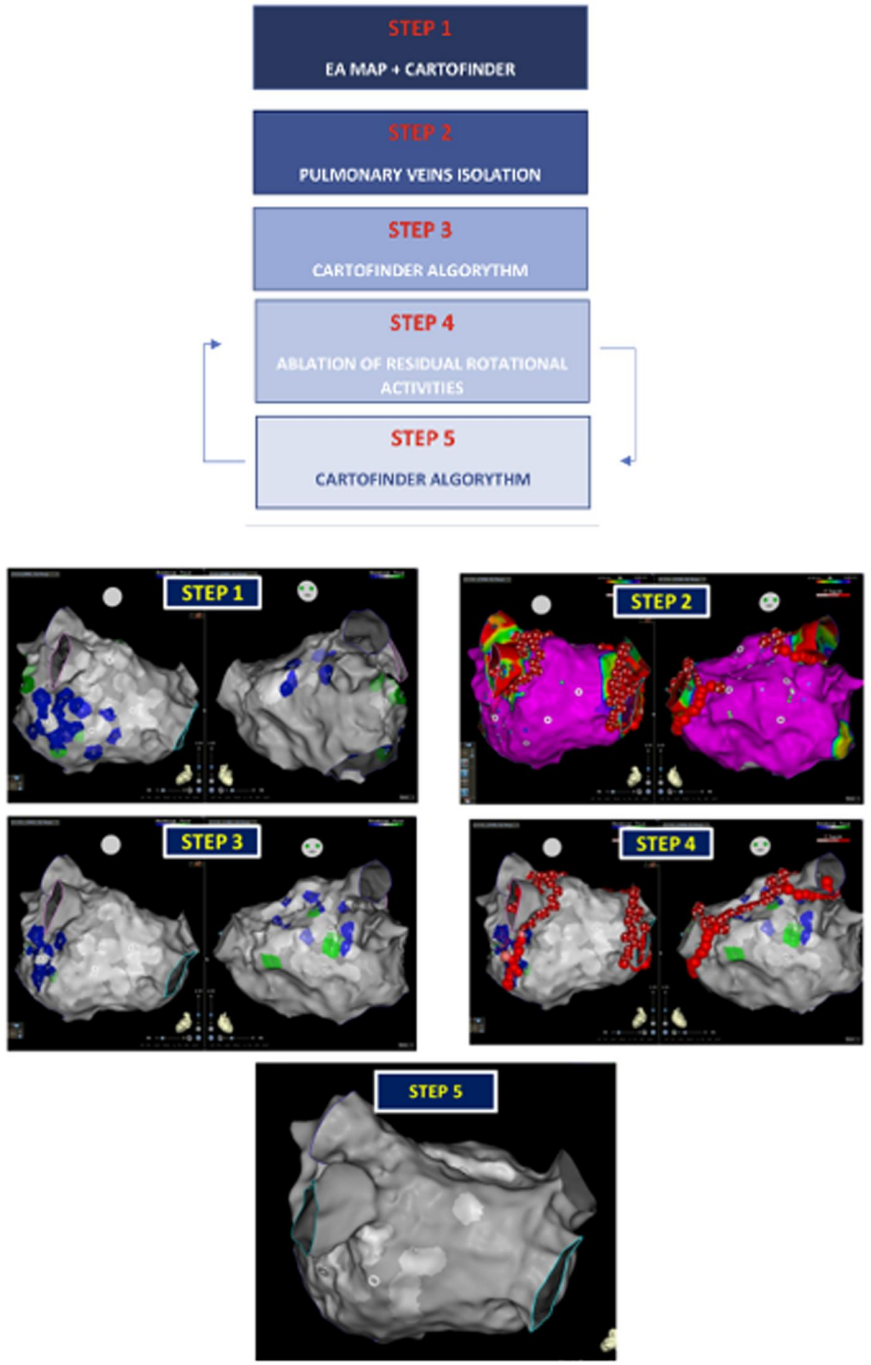
Illustration of the study protocol workflow. Restoration of the steps after pulmonary vein isolation would be considered the final step for the patient. STEP 4 and STEP 5 were repeated until the disappearance of any residual rotational activity.

Our workflow consisted of (Figure 1)

- STEP 1: Electroanatomical (EA) reconstruction of the LA using CARTO 3 with the FINDER algorithm during AF to create a bipolar map [range 0.05–0.24 mV (24)] and to identify RotAct. The operator was blinded to the CARTO Finder EA map.
- STEP 2: PVI was performed using Smart Touch, Smart Touch SF, or QDOT ablation catheters (*Biosense Webster*). When Smart Touch or Smart Touch SF were used, an ablation index with a target value of 500–550 on the anterior portion of the pulmonary veins and 380–400 on the posterior part were used. When QDOT Micro was used, PVI was carried out in QMODE plus modality (power: 90 Watt; Time of radiofrequency: 4 s; inter-lesion distance: 6 mm in posterior wall and 4 mm in anterior wall). PVI was confirmed at the end of STEP 2 mapping PVs with Pentaray Catheter (*Biosense Webster*).
- STEP 3: LA was newly mapped with the CARTO Finder algorithm with the aim of evaluating any residual RotAct.
- STEP 4: In the presence of residual RotAct, additional ablation lines were created to intersect the RotAct areas, connecting them to existing scar tissue or anatomical boundaries.
- STEP 5: a subsequent remap of the atrium was performed to confirm RotAct disappearance.

STEP 4 and STEP 5 were repeated until the elimination of all RotAct.

SR restoration at any point of the workflow was considered as the termination of the ablation procedure.

In case of persistence AF at the end of the procedure, an electrical cardioversion was carried out.

### Ablation procedure in the control group

Consecutive patients included in the control group had AF at the time of the procedure. Traditional fluoroscopic views and CARTO3 EAM (Electro Anatomic Mapping) (Biosense Webster, Irvine, CA) were used in all cases. The procedure was performed via both the right and left femoral veins.

A decapolar electrode catheter was positioned in the coronary sinus [Decanav (BiosenseWebster, Irvine, CA). Via a transseptal puncture or PFO, LA access was obtained. An ablation catheter was then inserted in the LA. EAM reconstruction was performed in every patient using Pentaray mapping catheter. All patients underwent PVI and extensive ablation (posterior wall, roof, mitral isthmus, and anterior wall) at the physician’s discretion, based on the presence of significant substrate remodeling and the persistence of AF after PVI. This approach was chosen given that current guidelines lack a clear indication for specific lesion schemas beyond PVI. In case of persistence of AF at the end of the procedure, an electrical cardioversion was carried out.

PVI was confirmed with a final LA EAM in the sinus rhythm, with the entrance block confirmed by the absence of EGMs in the PVs and the exit block confirmed by the absence of conduction of paced impulses from the inside of PVs to the LA. A linear lesion block was confirmed by positioning the ablator catheter tip to a side of the ablation line and then performing a LAT map showing the latest atrial activation on the opposite side of the linear lesion.

### Pharmacological therapy management

Following current recommendations from the ESC guidelines (1), the ablation procedure was performed without interruption of oral anticoagulation.

In case of preprocedural antiarrhythmic therapy, the AADs were stopped 5 days before the ablation procedure. The use of AADs after the ablation procedure was based on physician discretion.

### Subgroup analysis

The population was first divided into two groups:

– Group I (GI) represented patients without structural heart disease;
– Group II (GII) included patients with structural heart disease [coronary artery disease (CAD), valvular disease of moderate severity, hypertrophic cardiomyopathy (HCM), or dilatated cardiomyopathy (DCMP)].

Subsequently, patients were divided into two groups considering the presence or absence of RotAct at STEP 1: Group R (+) and Group R (−).

Therefore, we evaluated

– the presence, number, and distribution of RotAct before PVI (STEP 1) in the LA;
– The influence of PVI on the disappearance or *de novo* appearance of RotAct in the LA in both groups R(+) and R(−) (STEP 3);
– Re-assessed results dividing the patients into those with or without structural heart disease (GI and GII);
– The percentage of patients with spontaneous restoration of SR during the procedure in the whole population and subgroups;
– The persistence of SR at the end of the follow-up in the whole population, which was then compared between the groups with and without structural heart disease and the groups with and without RotAct before PVI, excluding possible confounding factors like the use of AADs.

### Statistical analysis

Categorical variables are presented as absolute numbers and relative percentages, while continuous variables with a normal distribution are presented as mean ± standard deviation. Categorical variables have been analyzed with a Chi squared test, Fisher test, and McNemar test while continuous variables have been analyzed with Welch *t*-test. Survival free from arrhythmia relapse was analyzed with a Kaplan–Meier graph, compared by log rank test. The presence of a difference with *p*value <0.05 was considered statistically significant.

Statistical analyses were performed utilizing the SAS Analytics Software 7.1 (64 bit).

## Results

### Study population

76 patients were consecutively enrolled during the study in the main study group. The main clinical data are listed in Table 1.

**Table 1:** General population characteristics divided by subgroup: group I and group II and between the presence or absence of rotational activity at STEP 1 (group R+ and R−).

Population characteristics	GENERAL (*n* = 76)	GROUP I (*n* = 49)	GROUP II(*n* = 27)	*P* value	R+ (*n* = 29)	R− (*n* = 47)	*P* value
Age	63 ± 8	63 ± 8	65 ± 9	0.32	64 ± 8	63 ± 8	0.85
Male	61 (81%)	40 (52%)	21 (28%)	0.069	25 (33%)	36 (47%)	0.3
Weight (kg)	89 ± 20	90 ± 8	89 ± 23	0.82	87 ± 13	90 ± 23	0.48
Height (cm)	174 ± 12	173 ± 0.4	175 ± 8	0.68	176 ± 7.5	172.5 ± 14	0.17
BMI	31.1 ± 17	32.1 ± 9	29 ± 6	0.51	28.4 ± 4	32.2 ± 11	0.3
Hypertension	53 (70%)	33 (43%)	20 (26%)	0.93	20 (26%)	33 (43%)	0.61
Hyperthyroidism	2 (2.6%)	1 (1.3%)	1 (1.3%)	0.8	1 (1.3%)	1 (1.3%)	
Hypothyroidism	8 (10.5%)	4 (5.3%)	4 (5.3%)	0.7	4 (5.3%)	4 (5.3%)	0.76
Stroke	6 (8%)	4 (5.3%)	2 (2.6%)	0.8	2 (2.6%)	4 (5.3%)	0.75
Diabetes	10 (13%)	7 (9.2%)	3 (3.9%)	0.57	4 (5.3%)	6 (8%)	0.97
Heart Failure	9 (12%)	1 (1.3%)	8 (10.5%)	**0** **.** **01**	3 (3.9%)	6 (8%)	0.69
ECV	56 (74%)	37 (49%)	19 (25%)	0.61	21 (28%)	35 (46%)	0.4
AADs Ic	17 (22%)	12 (15.8%)	5 (6.6%)	0.53	7 (9.2%)	10 (13%)	0.47
AADs III	29 (38%)	18 (24%)	11 (14.5%)	0.86	9 (12%)	20 (26%)	0.17
Beta Blockers	50 (66%)	29 (38%)	21 (28%)	0.29	20 (26%)	30 (39.5%)	0.88
Digoxin	7 (9.2%)	4 (5.3%)	3 (3.9%)	0.81	4 (5.3%)	3 (3.9%)	0.33
LA Area ind.	13.3 ± 3	14 ± 4	10.3 ± 3	0.78	13 ± 5	12.3 ± 3	0.37
LA Volume ind.	42.1 ± 5	41 ± 21	44 ± 24	0.43	38 ± 16	44 ± 25	0.54
EF%	55.1 ± 2	58.4 ± 7.6	52.9 ± 10	**0** **.** **02**	53 3 16	57 25	0.50
Moderate MR	14 (18%)	0	14 (18%)	**<0** **.** **01**	4 (5.3%)	10 (13%)	0.21
Moderate AR	3 (3.9%)	0	3 (3.9%)	0.03	0	3 (3.9%)	0.12

AADs, anti-arrhythmic drugs; AR, aortic regurgitation; BMI, body mass index; ECV, electrical cardioversion; EF, ejection fraction; LA, left atrium; MR, mitral regurgitation.

56 patients were included in the control group. The main clinical characteristics are listed in Table 2. Patients in the control group were significantly younger (*p* = 0.03) and had less history of hypertension (*p* = 0.01). Median CHADS-VA was 2 (IQ 0–5). All patients in the control group underwent PVI during the index procedure. 10 patients underwent adjunctive ablation: 4 posterior box ablation, 4 LA roof, 2 mitral isthmus, and 2 anterior wall.

**Table 2:** General population characteristics in the study group and control group.

Population characteristics	Study Group (*n* = 76)	Control Group (*n* = 56)	*P* value
Age	63 ± 8	60 ± 5.9	**0** **.** **03**
Gender (Male)	61 (81%)	44 (78.6%)	0.8
Weight (kg)	89 ± 20	89 ± 16.7	0.97
Height (cm)	174 ± 12	177 ± 5.9	0.07
BMI	31.1 ± 17	27.7 ± 8.3	0.43
Hypertension	53 (70%)	28 (51.9%)	**0**.**01**
Dysthyroidism	10 (13.1%)	6 (10.7%)	0.16
Stroke	6 (8%)	4 (7.1%)	0.83
Diabetes	10 (13%)	4 (7.1%)	0.24
Heart Failure	9 (12%)	6 (10.6%)	0.79
ECV	56 (74%)	42 (75%)	0.84
AADs Ic	17 (22%)	26 (46.4%)	**0**.**03**
AADs III	29 (38%)	24 (42.8%)	0.38
Beta Blockers	50 (66%)	24 (42.8%)	**0**.**013**
Digoxin	7 (9.2%)	8 (14.2%)	0.32
LA Area ind.	13.3 ± 3	12.1 ± 1.4	0.27
LA Volume ind.	42.1 ± 5	40.2 ± 5.4	0.42
EF%	55.1 ± 2	58.4 ± 7.6	0.95
Moderate MR	14 (18%)	9 (16.1%)	0.84
Moderate AR	3 (3.9%)	2 (3.5%)	0.91

AADs, anti-arrhythmic drugs; AR, aortic regurgitation; BMI, body mass index; ECV, electrical cardioversion; EF, ejection fraction; LA, left atrium; MR, mitral regurgitation.

### Ablation procedure

**Figure 2:**
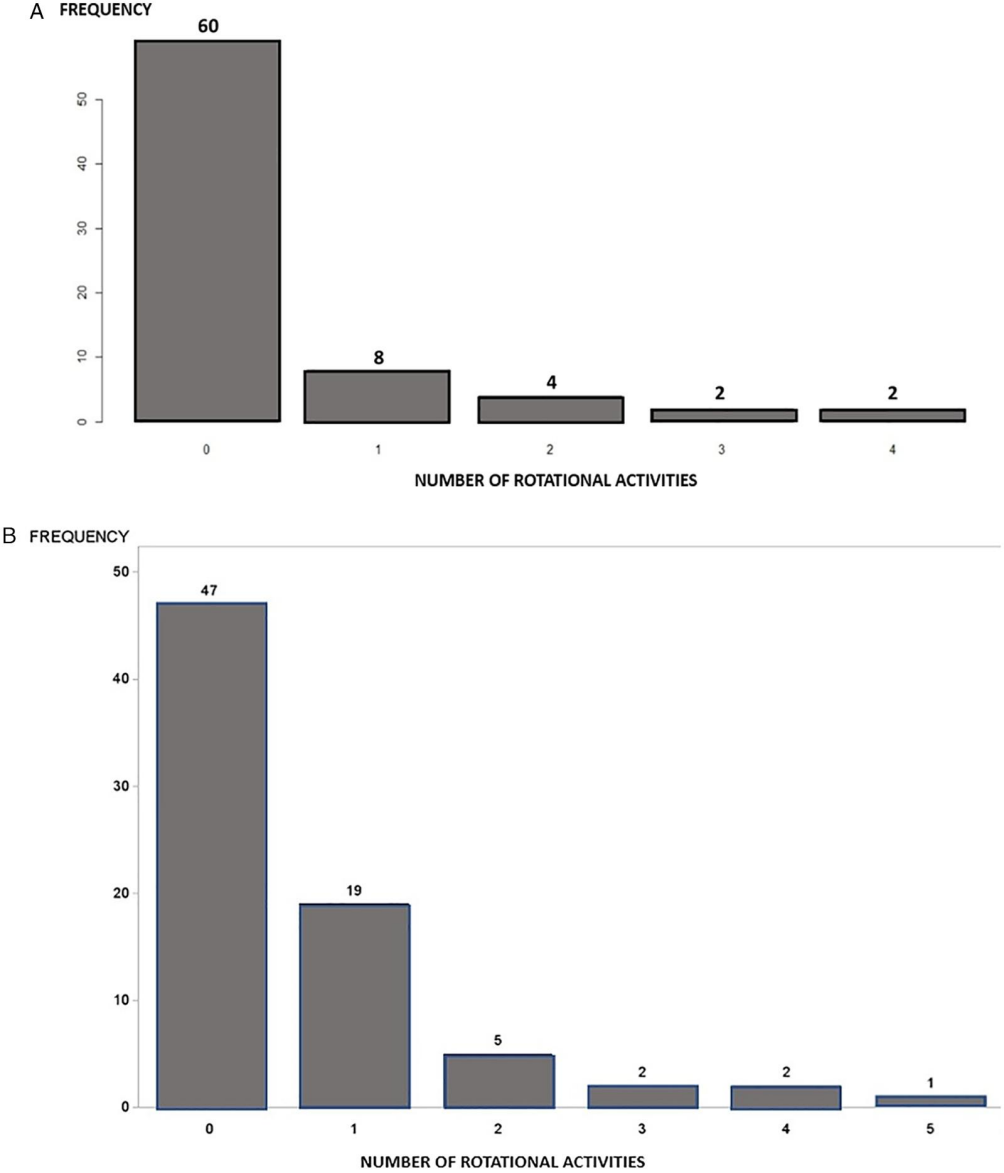
Number of rotational activities in the study group. **(A)** Number of rotational activities in the study patients identified at STEP 1. **(B)** Number of rotational activities in the study patients identified at STEP 3.

In STEP 1, 29 of the 76 total patients (38%) exhibited RotAct [group R(+)]. The number and distribution of RotAct are presented in Figures 2A, 4 respectively. No relevant clinical difference between group R(+) and R(−) patients was found (Table 2).

PVI was achieved in all the patients and all of them completed the workflow as planned.

**Figure 3:**
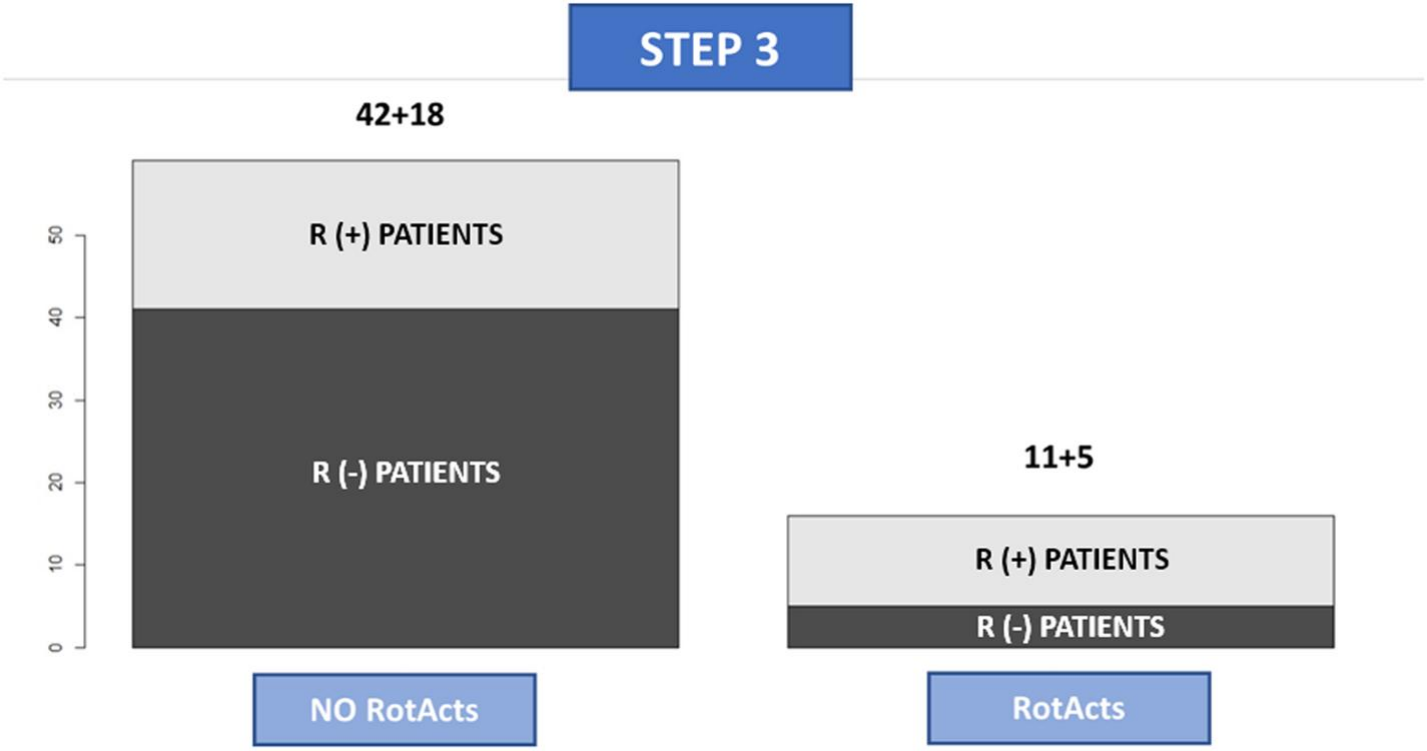
Absence and presence of rotational activity at STEP3 mapping. The first column is composed of patients without rotational activity after PVI, while the column on the right represents patients having rotational activity after PVI. The dark grey represents patients belonging to the group R- at STEP 1 while soft grey represents patients belonging to group R + at STEP 1.

PVI resulted in a significant variation in the number and distribution of RotAct (***p*-value = 0.01**). At STEP 3, RotAct remained in 11 out of 29 group R(+) pts (37%) and appeared *de novo* in 5 out of 47 groups of R(−) patients (10.6%) (Figure 3; Table 3).

**Figure 4:**
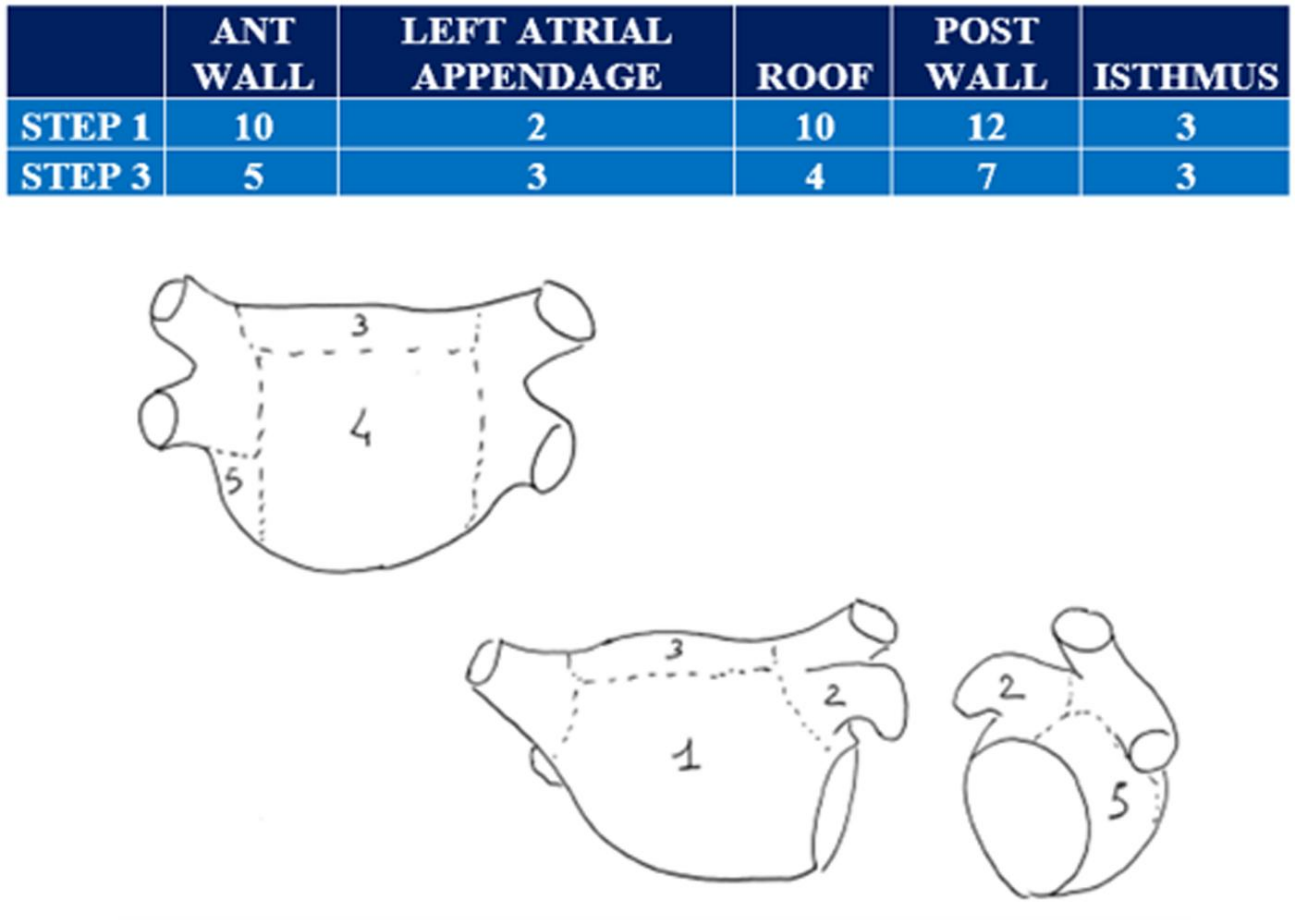
Left atrium localization of rotational activities at STEP 1 (first row) and at STEP 3 (second row). Illustration of the classification of the different segments of the left atrium: Anterior wall (1); Left Atrium Appendage (2); Roof (3); Posterior Wall (4); and Isthmus (5).

**Table 3:** Group distribution of rotational activities before and after PVI (STEP1 and STEP3 respectively).

Rotational Activities STEP 1	Total	GI	GII
R+	**29**	**22**	**7**
Group	**Tot STEP 3**	**GI**	**GII**
R+	**11**	**9**	**2**
**R-**	**5**	**4**	**1**

GI, absence of structural heart disease; GII, presence of structural heart disease; PVI, pulmonary vein isolation; R+, presence of rotational activity before PVI; R-, absence of rotational activity before PVI.

The number of RotAct at STEP 3 for each patient is presented in Figure 2B.

RotAct localizations at STEP 1 and STEP 3 are listed in Figure 4.

Complete abolition of RotAct was achieved in all patients after STEP 4.

Mean procedural time was 138 min (IQ 131–156 min).

No procedural complications were seen.

### Subgroup analysis

G I included 49 patients (65%) while G II included 27 patients (35%). G II presented CAD in 2 patients (7%), HCM in 3 patients (11%), DCM in 9 patients (34%), and moderate valvular disease in 13 patients (48%).

RotAct at STEP 1 was found in 22 out of 49 patients in G I (44.9%) and in 7 patients out of 27 in G II (25.9%), as reported in Table 3, without significant difference between the two groups (*p* = 0.14).

At STEP 3, residual RotAct was present in 13 out of 22 patients in G I and 3 out of 7 patients in G II (Table 3), without significant difference between the two study groups (*p* = 0.18). The number of RotAct identified for each patient at STEP 3 is presented in Figure 2B.

### Follow-up

**Figure 5:**
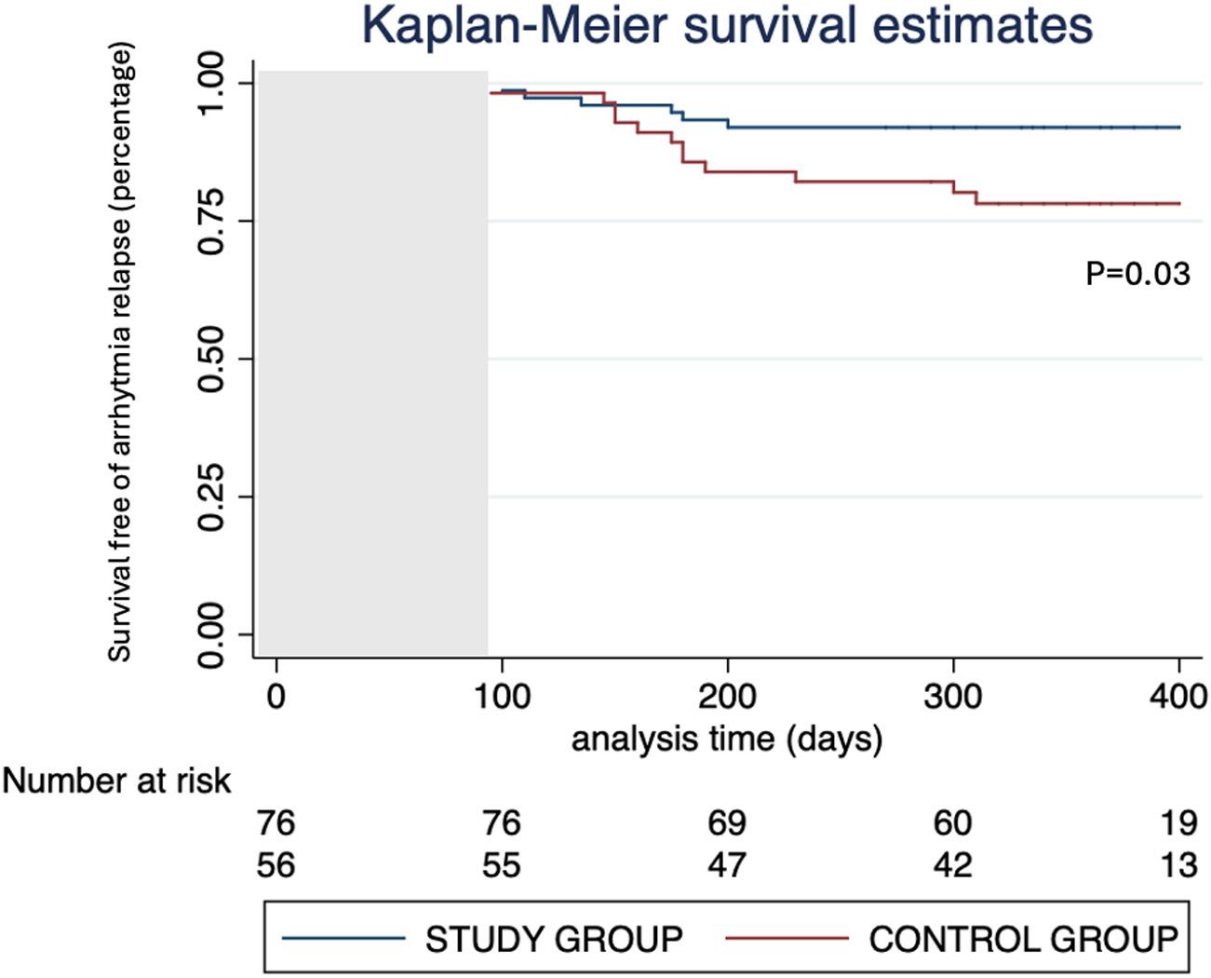
Kaplan–Meier graph of survival free from arrhythmia relapse in the study and control group, with a 3-month blanking period.

Follow-up lasted 13 ± 6 months. 5 out of 76 patients did not complete follow-up. 13 out of 71 evaluated patients (18.3%) presented relapses during the first three months after the ablation procedure. At the end of the follow-up, 6 out of 71 patients (8.5%) experienced AF recurrency, while 65 out of 71 patients (91.5%) maintained stable SR (*p* = 0.03, Figure 5).

Of the 6 patients with AF recurrence during the follow-up, all patients belonged to G I. 1 out of 6 patients belonged to R(+) and 5 out of 6 patients belonged to R(−). At STEP3, 2 out of 6 patients showed RotAct, 1 of which presented such activity at STEP 1 and 1 of which developed new RotAct.

In patients without relapses, 40 out of 65 (61.5%) belonged to GI while the remaining 25 (38.4%) belonged to GII; 38 patients (58%) belonged to R- while 27 patients (41.5%) belonged to R+.

At the end of follow-up, 42 out of 71 patients (59%) were on AADs (Table 4).

**Table 4:** Distribution of patients with and without relapses during follow-up in GI, GII, R+, R−; AADs+ and AADs−.

Recurrences	GI	GII	R+	R−	AADs+	AADs-
Yes	6	0	1	5	5	1
No	40	25	27	38	37	28
AADs	GI	GII	R+	R-		
42	24	18	15	27		

AADs, Anti-arrhythmic drugs; GI, absence of structural heart disease; GII, presence of structural heart disease; R+, presence of rotational activity before PVI; R-, absence of rotational activity before PVI.

During the follow-up period, no atypical atrial flutter was noted.

1 patient (3.5%) in group R(+) and 5 (11.6%) in group R(−) experienced AF relapse during follow-up, without significant difference between the groups (*p* = 0.21).

### Control group comparison

The control group included 56 patients with a mean follow-up time of 13 ± 5 months. At the end of the follow-up, 12 (21.4%) patients presented significant arrhythmia relapse (9 AF and 2 atypical flutter), a significant higher rate than in the study group (21.4% vs. 8.5%, *p* = 0.025). 27 patients (48.2%) were on AADs at the end of the follow-up. 10 (17.8%) patients in the control group underwent a re-do ablation procedure.

In the rotational activity subgroups, both patients in group R(+) and group R(−) experienced reduced incidence [group R(+) vs. control 3.5% vs. 21.4%, group R(−) vs. control 11.6% vs. 21.4%] of arrhythmia relapse during follow-up compared to the control group [respectively significant *p* = 0.036 for group R(+) and not significant *p* = 0.18 for group R(−)].

## Discussion

Currently, AF ablation guidelines indicate PVI as the standard procedure to treat AF, but the optimal ablation strategy has not been clarified in the non-paroxysmal AF population (1). However, the success rate of PVI in persAF is consistently lower compared to paroxysmal AF (20–22, 24), even when PFA is used (17–19), implying a different etiopathogenesis and a more extensive left atrial remodeling (7–9). Different strategies have been proposed to improve the ablation outcome in persAF including CFAE ablation (10, 11), linear left atrium lesions (12, 13), and/or the creation of a posterior box (14, 15) presenting conflicting results. The STAR AF II trial was designed to evaluate the efficacy of these different techniques. The results showed no statistically significant difference in terms of success rates when combining linear and CFAE ablation strategies with PVI (16).

In the search for alternative etiopathogentic mechanisms to explain poor ablation outcomes in pers AF, Narayan et al. investigated the efficacy of RotAct ablation (20). The author reported encouraging results although these were not reproduced by other authors (21, 22, 24).

Biosense Webster created the CARTO Finder Module, which is able to detect rotational and focal atrial activity (25).

We decided to design our prospective multicenter study using the CARTO Finder software to evaluate the presence and distribution of RotAct in persAF, as well as the influence of PVI on this activity, the impact of adding the ablation of RotAct areas to PVI in this group of patients, and the comparison of this population with a control group undergoing “standard” persAF ablation protocol.

The main results of the study are as follows:

– RotAct was identified in 29 out of 76 (38%) persAF patients before the PVI (STEP1). This percentage was lower than that of previous studies (25, 26) even if it is difficult to compare these results considering the different mapping algorithms used.
– RotAct are localized in the entire LA wall without any statistically significant differences regarding the different sites.
  In addition, no differences were identified in the general clinical characteristics (age, sex, previous use of antiarrhythmic drugs, previous history of electrical cardioversion, anatomical characteristics, etc.) in subjects with or without RotAct. Interestingly, a statistically significant difference in the presence and number of RotAct was not identified between the patients without (GI) or with (G II) structural heart disease. This result is surprising considering that RotAct is generally associated with a remodeling of LA. It can be speculated that the RotAct may also be due to functional modifications of electrical properties of LA regardless of underlying cardiopathy (27).
– On the other hand, PVI significantly modified the number and localization of RotActs (*p* value = 0.012). In fact, RotAct present at STEP 1 remained after PVI in only 11 out of 29 originally affected subjects (R+) while it appeared *de novo* in 5 patients belonging to the R- group. An explanation for these results may be that PVI modified the electrical wavefront propagation inside the LA, leading to the disappearance and/or *de novo* appearance of RotActs (27). To reinforce these results, it should be mentioned that, to avoid a potential bias, the operator was blinded to the results of the CARTO Finder mapping before the isolation of PV in order to not affect the operator's choice regarding the PV ablation schema that may include rotors localized in proximity to PV ostium.
– The goal of the ablation procedure was the PVI and the complete elimination of all RotActs at the end of the procedure. This goal was achieved in all the patients, confirming its feasibility.
– Noteworthy, no complications were noted and, particularly, no atypical atrial flutter was seen in the follow-up. This can be attributed to the lesser amount of linear lesions in both study groups when compared to previous studies (20–22, 24, 28–31). This result can be explained by the low rate of patients who underwent linear lesion ablation both in RotAct and control groups and the fact that the operators were highly experienced.
– The results of the study show that this tailored approach presents a very high success rate in a long-term follow-up period. In fact, 65 out of 71 patients (91%) who completed a mean follow-up of 13 ± 6 months maintained stable sinus rhythm. 42 out of these 71 patients (59%) were still on AADs due to the clinician's preference with a similar distribution between the groups.

In contrast to the STAR-AF study results (16), in our series the success rate was higher. Such a difference may have several explanations. First it could be due to the technological advancement, considering that in our study ablation index guided the RF delivery (32) and/or, in some patients, very high-power short duration RF delivery was used. A second explanation may also be the ability of CARTO Finder to identify RotAct and consequently to tailor the best ablation strategy to each patient. The adjunctive linear lesion guided by the mapping procedure seems to increase the ablation effectiveness while reducing iatrogenic complications, confirmed by the absence of atypical flutter in the follow-up.

Although good results of the ablation of RotActs in persAF have already been highlighted in other studies (21, 24, 28, 29, 33, 34), some studies reported poor results (22, 35) which were attributed either to an inadequate mapping and ablation strategy (36) or less experienced centers.

In this study, the proposed algorithm was developed to evaluate the impact of RotActs ablation in addition to PVI on AF freedom in patients with PersAF. This approach prolonged the procedural time due to a research-only step (STEP 1) that would not be required in routine clinical practice. In fact, assuming the efficacy of RotActs ablation on top of PVI, the extra time needed compared to PVI alone would be limited to the use of the Finder map at the end of PVI and eventual linear lesions.

The value of the present study, besides the success rate, is that it is the first report confirming the influence of PVI on RotAct and that demonstrates a user-friendly, widely available, and reproducible mapping technique.

A comparison with the control group of consecutive PersAF patients undergoing standard protocol (PVI and additional linear lesions according to the physician’s choice) showed a significant increase in survival free of relevant arrhythmia relapse (91.5% in the study group vs. 78.6% in the control group, *p* = 0.025). In addition, our protocol seems to reduce the need for adjunctive linear lesions by selecting the subgroup of patients who might benefit from it and consequently reducing the risk of atypical atrial flutter onset during follow-up.

Although these preliminary results are encouraging, further randomized studies in larger cohorts of patients are needed to validate the findings.

After completing this study, we decided in our lab to modify our daily practice, performing ablation of persAF starting with PVI and then continuing with atrial mapping to search for RotAct to eliminate them and increase the success rate in this population.

## Study limitations

The small sample size, the absence of randomization, the absence of multivariable analysis, and the involvement of only high-volume EP laboratories represent limits to our study and so it is not possible to establish with certainty whether the overall success rate can be limited to the additional linear ablation of RotActs or to confounder factors.

Furthermore, the study had additional limitations: mapping was not performed in the right atrium, and the protocol left the discontinuation of AAD after the initial three-month follow-up to the clinicians’ discretion. This led to a higher incidence of AAD use in the interventional group than in the control group, potentially confounding the results.

Considering that the present study aimed to both evaluate mapping and ablation outcome, we expected a longer procedural time that was balanced by cost-effectiveness.

The absence of a time-spaced second EA map of the left atrium using the Finder algorithm after the one performed in STEP 1 to confirm the persistence in number and distribution of RotActs in LA allowed us to hypothesize the role of PVI in determining a change in the number and distribution of RotActs but not to establish a certain cause-effect, for which more extensive studies on the subject are needed.

Operators were not blinded to the results of the LA voltage map and, when performing linear lesions in the LA to interrupt RotAct, may have considered the presence of adjacent low-voltage areas to guide the path of the ablation lines, representing a possible confounder.

Lastly, the follow-up by 24-Hour Holter ECG and occasional ECGs could possibly affect the detection of asymptomatic paroxysmal AF, which might have increased the perceived high success rate of the workflow proposed.

## Conclusions

The presence of RotAct in persAF accounts for 38% of patients regardless of the presence of structural heart disease.

PVI is able to modify the number and distribution of the RotAct in this population.

A tailored, combined strategy of PVI and RotAct ablation provided a high success rate in this group of patients in a long-term follow-up. The CARTO FINDER algorithm appears to be a promising tool to unveil the RotAct and guide its ablation after PVI.

## Data Availability

The raw data supporting the conclusions of this article will be made available by the authors upon reasonable request without undue reservation.

## Supplementary material

SUPPLEMENTARY VIDEO 1

Example of recording of Rotational Activity by CARTO-Finder software during ablation.
